# Facile Synthesis of Bis(indolyl)methanes Catalyzed by α-Chymotrypsin

**DOI:** 10.3390/molecules191219665

**Published:** 2014-11-27

**Authors:** Zong-Bo Xie, Da-Zhao Sun, Guo-Fang Jiang, Zhang-Gao Le

**Affiliations:** Department of Applied Chemistry, East China Institute of Technology, Nanchang 330013, China; E-Mails: zbxie@ecit.edu.cn (Z.-B.X.); anhuisdz@163.com (D.-Z.S.); gfjiang@ecit.cn (G.-F.J.)

**Keywords:** bis(indolyl)methanes, tandem protocol, biocatalysis, promiscuity, α-chymotrypsin

## Abstract

A mild and efficient method catalyzed by α-chymotrypsin was developed for the synthesis of bis(indolyl)methanes through a cascade process between indole and aromatic aldehydes. In the ethanol aqueous solution, a green medium, a wide range of aromatic aldehydes could react with indole to afford the desired products with moderate to good yields (from 68% to 95%) using a little α-chymotrypsin as catalyst.

## 1. Introduction

Bis(indolyl)methane and their derivatives are members of an important class of heterocyclic compounds that display diverse biological properties, and can act as a selective colorimetric sensor for F^−^ (or HSO_4_^−^) and also as a highly selective fluorescent molecular sensor for Cu^2+^ [1,2]. They are of immense interest because of their wide spectrum of pharmacological properties, such as antibacterial activity [3], antiangiogenic activity [4], acting as cytotoxic agents [4] and tumor growth inhibitors [5]. In the past years, various methods were mentioned for the synthesis of bis(indolyl)methanes, generally, these compounds could be obtained by the cascade reaction between indole and aromatic (or aliphatic) aldehydes in the present of protic or Lewis acids, such as I_2_ [6], Ionic liquids [7], Fe(DS)_3_ [8], CeCl_3_·7H_2_O [9], AuCl [10], SBA-15/SO_3_H [11], TPPMS/CBr_4_ [12], PEG-supported dichlorophosphate [13], H_3_PW_12_O_40_ [14], zeolites [15]. In fact, the bis(indolyl)methanes could also be prepared from indole and benzyl alcohols [16,17]. Although bis(indolyl)methanes could be effectively synthesized via most of the above attempts in excellent yields, there are some drawbacks in the vast majority of the described methods including the use of expensive and toxic heavy metals, requirement of a stoichiometric amount of catalysts, complicated post-treatment process, and so on.

As an efficient, high selectivity and eco-friendly catalyst for the organic synthesis, enzymes have attracted much attention in the field of synthetic chemistry [18,19,20]. Especially, in recent years, some hydrolases have demonstrated high activity for unnatural substrates and alternative chemical transformations, namely, biocatalytic promiscuity, which provides a new tool for organic synthesis and largely extends the application of enzymes [21,22]. Enzyme promiscuity has been widely used in multiple types of organic reactions, such as C-C [23], C-N [24] and C-S [25] bond-formation reactions. Recently, Lin and coworkers [26] reported the synthesis of bis(indolyl)methanes catalyzed by PPL (Lipase from porcine pancreas), but the results are waiting to be lifted. Therefore, effective, eco-friendly and sustainable biocatalytic methods are still to be explored.

**Scheme 1:**
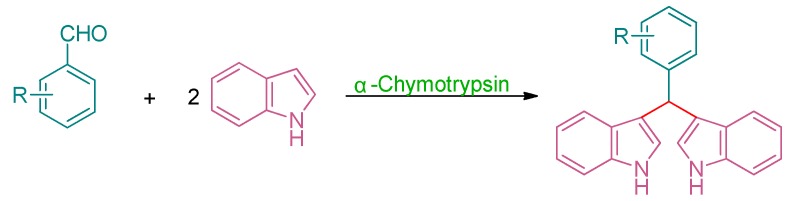
α-Chymotrypsin-catalyzed tandem reaction of indole and aldehydes.

Thus, as a part of our continuing interest in green chemistry and enzyme promiscuity, we wish to report the protease-catalyzed cascade reaction between indole and aromatic aldehydes (Scheme 1). Here, α-chymotrypsin was selected as the biocatalyst, and a series of aromatic aldehydes could react with indole to afford the corresponding bis(indolyl)methanes in ethanol aqueous solution, which was obviously a green synthesis method.

## 2. Results and Discussion

Based on our previous research, initial efforts were performed in 40% ethanol solution using 4-nitrobenzaldehyde and indole as a model reaction and some hydrolases were investigated to screen the optimal catalyst. As shown in Table 1, α-chymotrypsin displayed the best catalytic activity and gave the corresponding product in 77% yield (Table 1, entry 8), pepsin also provided 51% yield (Table 1, entry 7). However, the other tested hydrolase, such as Neutral protease, Alkaline protease, Papain, Acylase I from *Aspergillus melleus* (Acylase I), PPL and Amano Lipase M from *Mucor javanicus* (Amano Lipase M) only provided fewer products (Table 1, entries 1–6). At the same time, non-enzyme protein BSA (Bovine serum albumin) (Table 1, entry 9) and denatured α-chymotrypsin (Table 1, entry 10) were also used as catalysts, while both showed inferior catalytic ability, and gained similar results with the blank control reaction (Table 1, entry 11). These results confirmed that the catalytic activity of α-chymotrypsin for the tandem reaction did not arise from unspecific amino acids, and its tertiary structure played a great role in this enzyme-catalyzed reaction. 

**Table 1:** Catalytic activities of different enzymes *^a^*. 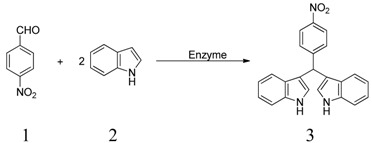

Entry	Enzyme	Yield *^b^* (%)
1	Neutral Protease	9
2	Alkaline protease	15
3	Papain	22
4	Acylase I	10
5	PPL	29
6	Amano Lipase M	6
7	Pepsin	51
8	α-Chymotrypsin	77
9	BSA	5
10	Denatured α-chymotrypsin *^c^*	5
11	No enzyme	4

*^a^* Conditions: 4-nitrobenzaldehyde 75.6 mg (0.5 mmol), indole 117.1 mg (1.0 mmol), enzyme 10 mg, ethanol 2 mL and deionized water 3 mL, at 40 °C for 24 h; *^b^* Isolated yield after column chromatography; *^c^* Pretreated with urea solution (8 mol/L).

**Figure 1:**
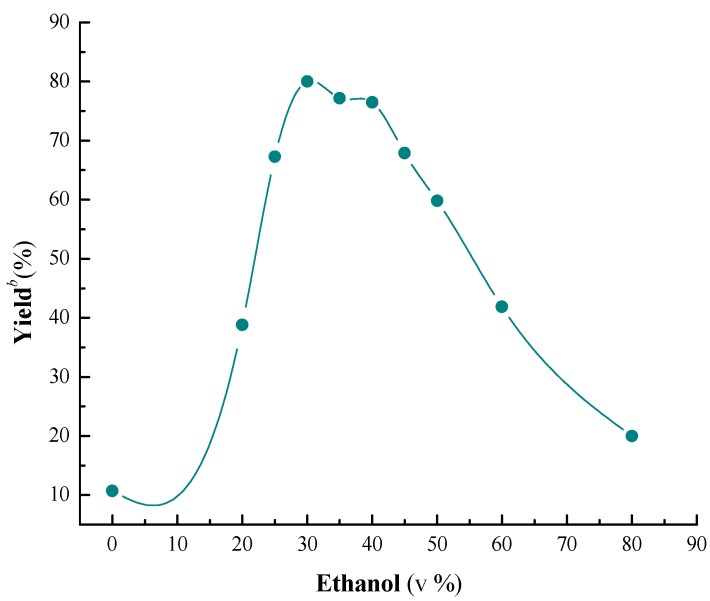
Influence of the ethanol content on the cascade reaction *^a^*.

*^a^* Conditions: 4-nitrobenzaldehyde 75.6 mg (0.5 mmol), indole 117.1 mg (1.0 mmol), α-chymotrypsin 10 mg, solvent 5 mL, at 40 °C for 24 h; *^b^* Isolated yield after column chromatography.

α-Chymotrypsin was selected as the best catalyst, the influence of the ethanol content was then investigated, because the reaction medium has been recognized as a significant factor for enzymatic reaction. The results are shown in Figure 1, it can be seen that the yield was significantly influenced by ethanol content, and 30% was selected as the optimum concentration. A possible reason may be that ethanol is conducive to the dissolution of the substrates; nevertheless, over much ethanol can lead α-chymotrypsin to lose activity. As a result, the yield was rising greatly by increasing the concentration of ethanol from 0% to 30% and reached the maximum at 30%, and then the yield decreased significantly with the rise of the concentration.

**Figure 2:**
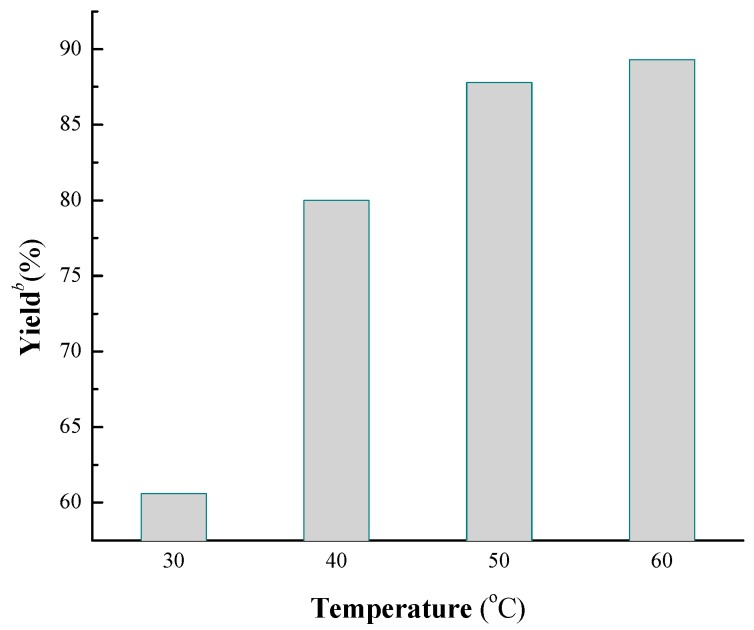
The influence of temperature on the cascade reaction *^a^*.

Temperature is another key influencing factor on the biocatalytic reaction, because of its effect on the biological activity of enzyme and the rate of the reaction. As can be seen from Figure 2, a yield of 88% was gained at 50 °C and 90% was gained at 60 °C. Though a slightly higher yield was obtained at 60 °C, 50 °C was chosen as the optimal temperature considering of the energy consumption and the inactivation of enzyme at high temperature for a long time.

*^a^* Conditions: 4-nitrobenzaldehyde 75.6 mg (0.5 mmol), indole 117.1 mg (1.0 mmol), α-chymotrypsin 10 mg, ethanol 1.5 mL and deionized water 3.5 mL, at a certain temperature for 24 h; *^b^* Isolated yield after column chromatography.

**Figure 3:**
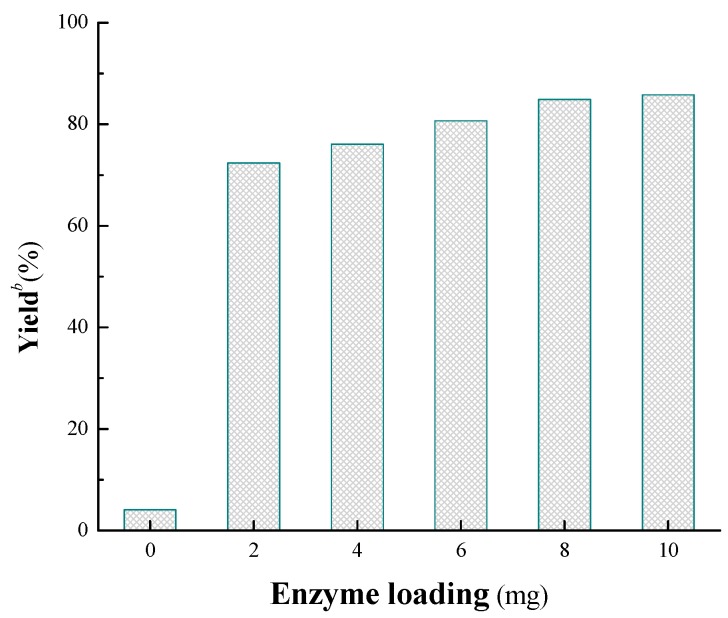
Influence of the enzyme loading on the cascade reaction *^a^*.

To further optimize the reaction conditions, the effect of enzyme loading on the α-chymotrypsin-catalyzed tandem reaction was investigated. As shown in Figure 3, only a little product was detected in the absence of enzyme, however, the yield was improved sharply when 2 mg α-chymotrypsin was loaded. After that, only a slight rising trend was appeared with the increase of catalyst dosage (2–10 mg), at last 8 mg was chosen as the best enzyme loading. However, it is particularly worth mentioning that the enzyme dosage is much lesser than most of the other reports about enzyme promiscuity, for example, 200 mg enzyme was used in a protease-catalyzed aldol reaction with 1.15 mL reaction medium [27]. 

With the optimized conditions in hand, more kinds of aromatic aldehydes were used to show the generality and scope of this enzymatic cascade reaction. The results are summarized in Table 2, it can be seen that a wide range of aromatic aldehydes can effectively react with indole to give the corresponding products, and the best yield of 95% has been obtained. However, the substituent in aromatic aldehyde has marked impact on the yield. Generally, aromatic aldehydes bearing an electron-withdrawing substituent, such as a nitro group, gave better results (Table 2, entries 1–7). However, some aldehydes with electron-donating groups, e.g., 4-hydroxybenzaldehyde and 4-methylbenzaldehyde showed lower activity, and moderate yields were gotten. However, beyond that, we also examined the reaction of aliphatic aldehydes with indole, however, the results were far from satisfactory. Caproaldehyde, one of the tested aliphatic aldehydes, only gave the best yield of 20% (data not given). Meanwhile, the tandem reactions about other heterocyclic compounds are proceeding.

*^a^* Conditions: 4-nitrobenzaldehyde 75.6 mg (0.5 mmol), indole 117.1 mg (1.0 mmol), α-chymotrypsin 0–10 mg, ethanol 1.5 mL and deionized water 3.5 mL, at 50 °C for 24 h; *^b^* Isolated yield after column chromatography.

**Table 2:** Investigation of the reactant scope of the α-chymotrypsin-catalyzed cascade reaction *^a^*. 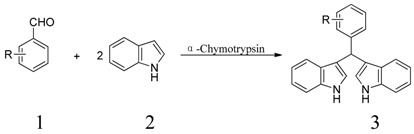

Entry	R	Product	Yield *^b^* (%)
1	4-NO_2_	3a	95
2	3-NO_2_	3b	92
3	2-NO_2_	3c	90
4	4-Cl	3d	95
5	2-Cl	3e	94
6	3-Br	3f	89
7	2-Br	3g	86
8	4-OH	3h	70
9	2-OH	3i	68
10	4-CH_3_	3j	71
11	4-OCH_3_	3k	75
12	4-OH, 3-OCH_3_	3l	79

*^a^* Conditions: aromatic aldehyde 0.5 mmol, indole 1.0 mmol, α-chymotrypsin 8 mg, ethanol 1.5 mL and deionized water 3.5 mL, at 50 °C for 32 h; *^b^* Isolated yield after column chromatography.

**Scheme 2:**
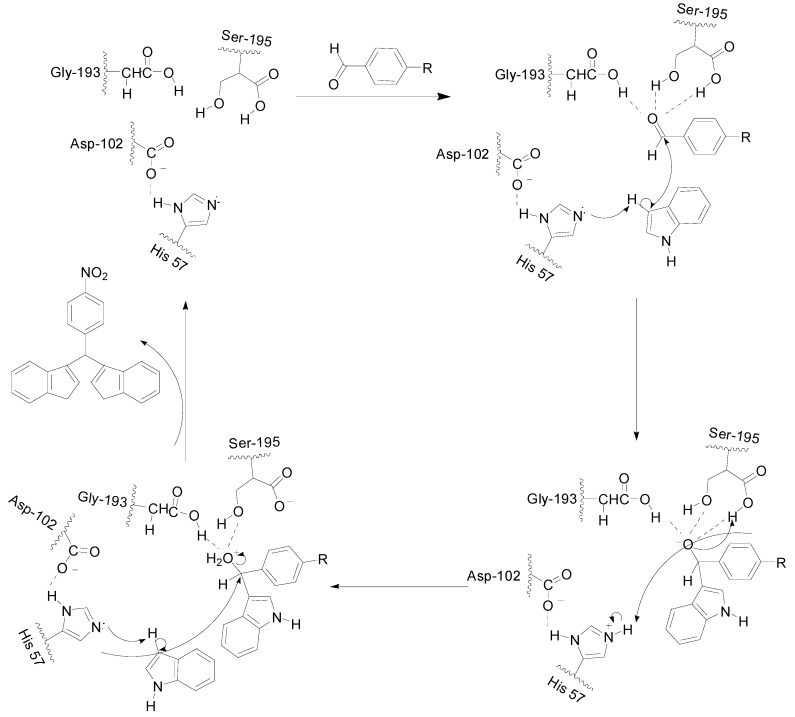
Proposed mechanism for α-chymotrypsin-catalyzed cascade reaction.

Finally, we attempt to propose the mechanism for the α-chymotrypsin-catalyzed cascade reaction. As one of the family of serine proteases, α-chymotrypsin is comprised of 245 amino acids and the catalytic triad is formed by His57, Asp102, and Ser195 [28,29,30]. According to this information, a mechanism of α-chymotrypsin-catalyzing such reaction was invisaged. As shown in Scheme 2, the aldehyde carbonyl bind to the oxyanion hole and be effectively activated [31,32]. His 57 may abstract the proton from the C-3 position of indole, allowing the indole as an available nucleophile to bind to aromatic aldehyde, Ser195, and Gly193 could stabilize the formation of an oxyanion by hydrogen-bonding with the carbonyl oxygen of the aromatic aldehyde. Subsequently, the released indolyl methanol and another molecule of indole are joined together in a similar fashion, which could lead to the formation of the product and liberated α-chymotrypsin.

## 3. Experimental Section 

### 3.1. General Information

All chemicals were purchased from commercial suppliers and the solvents were not redistilled before used. Amano Lipase M from *Mucor javanicus* (Amano Lipase M), Acylase I from *Aspergillus melleus* (Acylase I) and α-chymotrypsin were obtained from Sigma-Aldrich. Lipase from porcine pancreas (PPL), Bovine serum albumin (BSA), Alkaline protease, Neutral protease, Pepsin from bovine serum and Papain were obtained from Aladdin. 

^1^H and ^13^C-NMR spectra were recorded on a Bruker AV-400 spectrometer in CDCl_3_ or DMSO-d_6_. High-resolution mass measurements (HRMS) were recorded on a Thermo Fisher Scientific LTQ Orbitrap-XL mass Spectrometer. Chemical shifts were reported in ppm(δ). IR spectra were recorded on a Nicolet 380 FT-IR spectrophotometer. Melting points were measured using a WRS-1B Digital Melting Point Apparatus.

### 3.2. General Procedure for the Synthesis of Bis(indolyl)methane 

A mixture of 4-nitrobenzaldehyde (0.0756 g, 0.5 mmol, 1 equiv.), indole (0.1171 g, 1 mmol, 2 equivalent) and α-chymotrypsin (10 mg) in mixed solvents (3 mL water and 2 mL ethanol) was incubated at 50 °C and 260 r.p.m. for 24 h. After completion of the reaction (TLC), the products were extraction with 3 × 5 mL ethylacetate. Then, the combined organic layer was concentrated under reduced pressure to afford the crude product and purified by column chromatography on silica gel (PE:EtOAc = 9:1) to give the pure product. 

#### *3,3'-((4-Nitrophenyl)methylene)bis(1H-indole)* (**3a**)


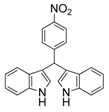


Red solid, mp: 219–220 °C; IR ν (cm^−1^) (KBr): 3455, 1637, 1506, 1456, 1414, 1341, 1101, 744; ^1^H-NMR (400 MHz, DMSO): δ 10.95 (s, 2H), 8.15 (d, *J* = 8.7 Hz, 2H), 7.61 (d, *J* = 8.7 Hz, 2H), 7.38 (d, *J* = 8.1 Hz, 2H), 7.30 (d, *J* = 7.9 Hz, 2H), 7.06 (t, *J* = 7.5 Hz, 2H), 6.96–6.78 (m, 4H), 6.04 (s, 1H); ^13^C-NMR (100 MHz, DMSO): δ 153.61, 146.26, 137.10, 129.93, 124.35, 123.87, 121.58, 119.39, 118.91, 112.07, 40.66, 40.45, 40.24, 40.03, 39.83, 39.62, 39.41; HRMS (EI) *m/z* calcd for C_23_H_16_O_2_N_3_ [M−H]^−^ 366.12480, found: 366.12896.

#### *3,3'-((3-Nitrophenyl)methylene)bis(1H-indole)* (**3b**)


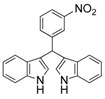


Red solid, mp: 86–87 °C; IR ν (cm^−1^) (KBr): 3411, 1637, 1508, 1456, 1417, 1339, 1094, 744; ^1^H-NMR (400 MHz, DMSO-*d*_6_): δ 10.94 (s, 2H), 8.17 (s, 1H), 8.07 (d, *J* = 8.0 Hz, 1H), 7.84 (d, *J* = 8.0 Hz, 1H), 7.58 (t, *J* = 8.4, 16.0, 7.6 Hz, 1H), 7.37 (d, *J* = 8.0 Hz, 2H), 7.30 (d, *J* = 8 Hz, 2H), 7.06 (t, *J* = 7.2, 14.8, 7.6 Hz, 2H), 6.88 (m, 4H), 6.07 (s, 1H).

#### *3,3'-((2-Nitrophenyl)methylene)bis(1H-indole)* (**3c**)


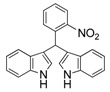


Red solid, mp: 140–142 °C; IR ν (cm^−1^) (KBr): 3419, 1636, 1560, 1523, 1454, 1417, 1352, 1096, 744; ^1^H NMR (400 MHz, DMSO): δ 10.93 (s, 2H), 7.88 (d, *J* = 7.9 Hz, 1H), 7.56 (t, *J* = 7.5 Hz, 1H), 7.46 (t, *J* = 7.3 Hz, 1H), 7.40 (d, *J* = 7.7 Hz, 1H), 7.36 (d, *J* = 8.1 Hz, 2H), 7.21 (d, *J* = 8.0 Hz, 2H), 7.06 (t, *J* = 7.5 Hz, 2H), 6.89 (t, *J* = 7.4 Hz, 2H), 6.78 (s, 2H), 6.40 (s, 1H).

#### *3,3'-((4-Chlorophenyl)methylene)bis(1H-indole)* (**3d**)


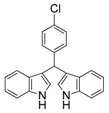


Red solid, mp: 75–76 °C; IR ν (cm^−1^) (KBr): 3417, 1637, 1486, 1455, 1416, 1338, 1092, 743; ^1^H-NMR (400 MHz, DMSO): δ 10.86 (s, 2H), 7.39–7.33 (m, 4H), 7.31 (d, *J* = 8.5 Hz, 2H), 7.27 (d, *J* = 7.9 Hz, 2H), 7.04 (t, *J* = 7.5 Hz, 2H), 6.87 (t, *J* = 7.5 Hz, 2H), 6.83 (s, 2H), 5.87 (s, 1H).

#### *3,3'-((2-Chlorophenyl)methylene)bis(1H-indole)* (**3e**)


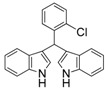


Red solid, mp: 71–72 °C; IR ν (cm^−1^) (KBr): 3414, 1627, 1458, 1417, 1338, 1093, 744; ^1^H-NMR (400 MHz, CDCl_3_): δ 7.93 (s, 2H), 7.64 (d, *J* = 7.8 Hz, 1H), 7.44 (d, *J* = 7.9 Hz, 2H), 7.38 (d, *J* = 8.1 Hz, 2H), 7.23 (s, 1H), 7.21 (s, 1H), 7.17 (s, 2H), 7.14–7.10 (m, 1H), 7.06 (d, *J* = 15.0 Hz, 2H), 6.62 (s, 2H), 6.34 (s, 1H).

#### *3,3'-((3-Bromophenyl)methylene)bis(1H-indole)* (**3f**)


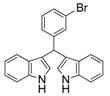


Red solid, mp: 104–106 °C; IR ν (cm^−1^) (KBr): 3440, 1636, 1454, 1416, 1342, 1096, 742; ^1^H-NMR (400 MHz, CDCl_3_): δ 7.97 (s, 2H), 7.49 (s, 1H), 7.41–7.34 (m, 5H), 7.31 (d, *J* = 21.5 Hz, 1H), 7.22–7.11 (m, 3H), 7.02 (t, *J* = 7.5 Hz, 2H), 6.65 (s, 2H), 5.85 (s, 1H). 

#### *3,3'-((2-Bromophenyl)methylene)bis(1H-indole)* (**3g**)


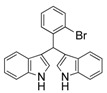


Red solid, mp: 76–77 °C; IR ν (cm^−1^) (KBr): 3416, 1637, 1457, 1416, 1339, 1094, 1017, 743; ^1^H-NMR (400 MHz, CDCl_3_): δ 7.88 (s, 2H), 7.46–7.27 (m, 5H), 7.19 (s, 1H), 7.15 (d, *J* = 5.8 Hz, 1H), 7.13–7.06 (m, 2H), 7.05–7.00 (m, 1H), 6.95 (t, *J* = 7.5 Hz, 2H), 6.56 (s, 2H), 6.27 (s, 1H); HRMS (EI) *m/z* calcd for C_23_H_16_BrN_2_ [M−H]^−^ 399.05023, found: 399.05086.

#### *3,3'-((4-Hydroxylphenyl)methylene)bis(1H-indole)* (**3h**)


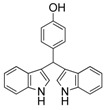


Red solid, mp: 195–196 °C; IR ν (cm^−1^) (KBr): 3416, 1617, 1509, 1454, 1416, 1338, 1218, 1166, 1095, 744; ^1^H-NMR (400 MHz, DMSO): δ 10.77 (s, 2H), 9.14 (s, 1H), 7.33 (d, *J* = 8.1 Hz, 2H), 7.26 (d, *J* = 7.9 Hz, 2H), 7.13 (d, *J* = 8.3 Hz, 2H), 7.02 (t, *J* = 7.5 Hz, 2H), 6.85 (t, *J* = 7.4 Hz, 2H), 6.77 (s, 2H), 6.65 (d, *J* = 8.4 Hz, 2H), 5.70 (s, 1H).

#### *3,3'-((2-Hydroxylphenyl)methylene)bis(1H-indole)* (**3i**)


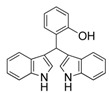


Red solid, mp: 255–256 °C; IR ν (cm^−1^) (KBr): 3419, 2924, 1620, 1516, 1483, 1455, 1420, 1195, 1096, 745; ^1^H-NMR (400 MHz, CDCl_3_): δ 7.96 (s, 2H), 7.34 (s, 1H), 7.31 (d, *J* = 3.4 Hz, 2H), 7.29 (s, 1H), 7.19 (s, 2H), 7.13 (d, *J* = 8.1 Hz, 2H), 7.09 (d, *J* = 7.0 Hz, 2H), 6.95 (t, *J* = 7.5 Hz, 2H), 6.80 (d, *J* = 3.8 Hz, 1H), 6.78 (d, *J* = 6.2 Hz, 1H), 6.70 (s, 2H), 5.93 (s, 1H).

#### *3,3'-((4-Methylphenyl)methylene)bis(1H-indole)* (**3j**)


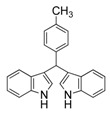


Red solid, mp: 99–101 °C; IR ν (cm^−1^) (KBr): 3421, 1635, 1512, 1454, 1416, 1339, 1095, 742; ^1^H-NMR (400 MHz, CDCl_3_): δ 7.91 (s, 2H), 7.39 (d, *J* = 8.0 Hz, 2H), 7.35 (d, *J* = 8.1 Hz, 2H), 7.22 (d, *J* = 7.9 Hz, 2H), 7.16 (t, *J* = 7.5 Hz, 2H), 7.08 (d, *J* = 7.7 Hz, 2H), 7.00 (t, *J* = 7.5 Hz, 2H), 6.66 (s, 2H), 5.85 (s, 1H), 2.31 (s, 3H).

#### *3,3'-((4-Methoxylphenyl)methylene)bis(1H-indole)* (**3k**)


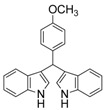


Pink solid, mp: 189–191 °C; IR ν (cm^−1^) (KBr): 3412, 1636, 1564, 1509, 1455, 1417, 1340, 1250, 1175, 1095, 1027, 744; ^1^H-NMR (400 MHz, CDCl_3_): δ 7.90 (s, 2H), 7.38 (d, *J* = 8.0 Hz, 2H), 7.34 (d, *J* = 8.1 Hz, 2H), 7.24 (d, *J* = 7.9 Hz, 2H), 7.16 (t, *J* = 7.4 Hz, 2H), 7.00 (t, *J* = 7.3 Hz, 2H), 6.81 (d, *J* = 8.6 Hz, 2H), 6.63 (s, 2H), 5.83 (s, 1H), 3.77 (s, 3H).

#### *4-(Di(1H-indol-3-yl)methyl)-2-methoxyphenol* (**3l**)


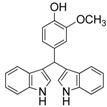


Red solid, mp 123–125 °C; IR ν (cm^−1^) (KBr): 3428, 1635, 1511, 1456, 1418, 1128, 744; ^1^H-NMR (400 MHz, CDCl_3_): δ 7.85 (s, 2H), 7.38 (d, *J* = 7.9 Hz, 2H), 7.30 (d, *J* = 8.1 Hz, 2H), 7.15 (t, *J* = 7.6 Hz, 2H), 6.99 (t, *J* = 7.5 Hz, 2H), 6.87 (s, 1H), 6.83–6.74 (m, 2H), 6.58 (s, 2H), 5.79 (s, 1H), 3.72 (s, 3H).

## 4. Conclusions

In conclusion, an effective, eco-friendly and convenient method for synthesis of bis(indolyl)alkanes was developed, which is the first example of such a protease-catalyzed reaction. α-Chymotrypsin, a promiscuous hydrolase, showed excellent catalytic activity for a series of substrates. As a new example of enzyme promiscuity, it is beneficial for expanding the application of biocatalysis in non-natural reactions. 

## Supplementary Materials

Supplementary materials can be accessed at: http://www.mdpi.com/1420-3049/19/12/19665/s1.
