# Supplementary material for: Facile Synthesis of Bis(indolyl)methanes Catalyzed by α-Chymotrypsin
Source: Molecules. 2014 Nov 27;19(12):19665–77. doi: 10.3390/molecules191219665 (PMC6271608; doi:10.3390/molecules191219665)

Supplementary Materials

3,3'-((4-Nitrophenyl)methylene)bis(1H-indole) (3a)

Figure S1. HRMS of compound **3a**.

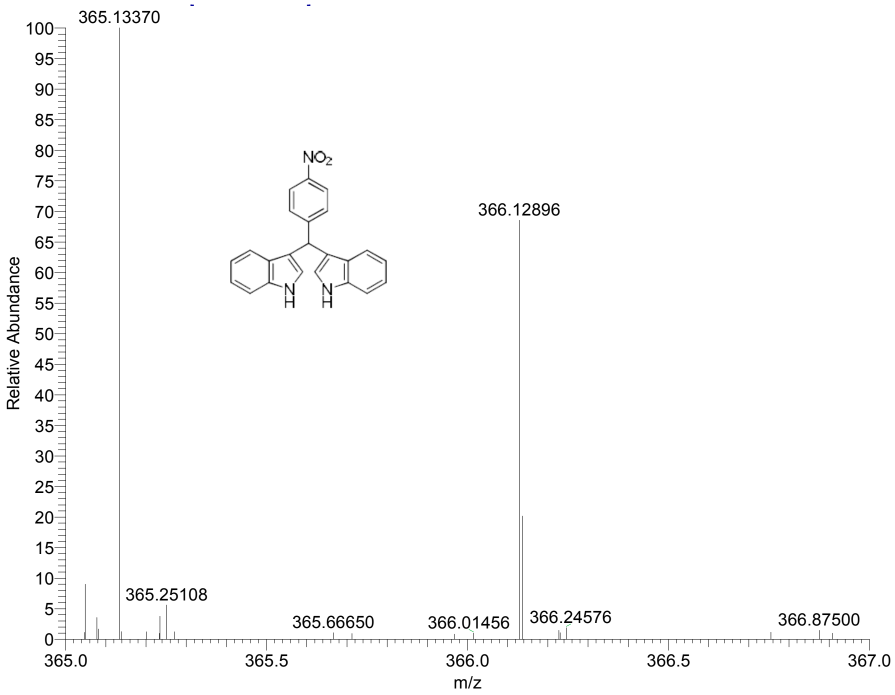

Figure S2. IR of compound **3a**.

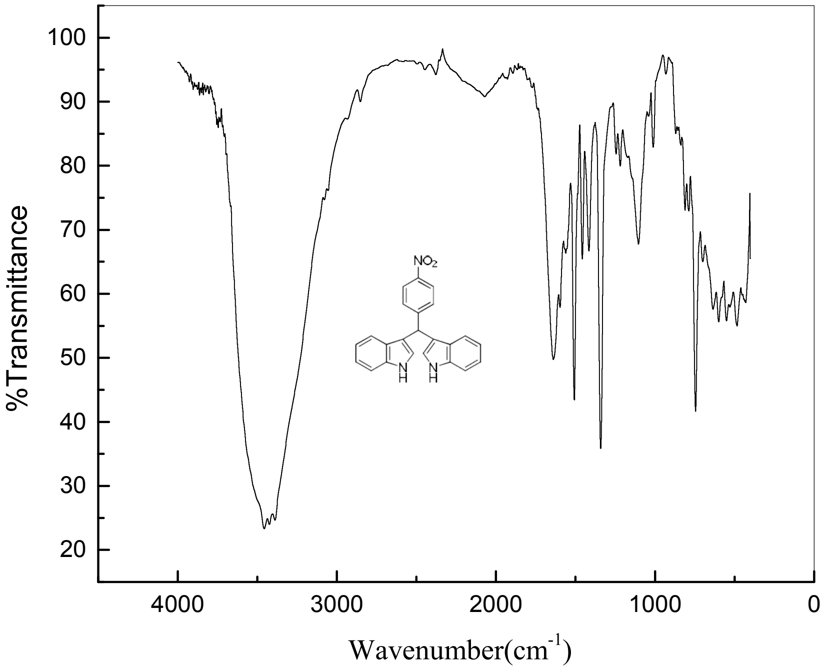

Figure S3. ^{1}H-NMR of compound **3a**.

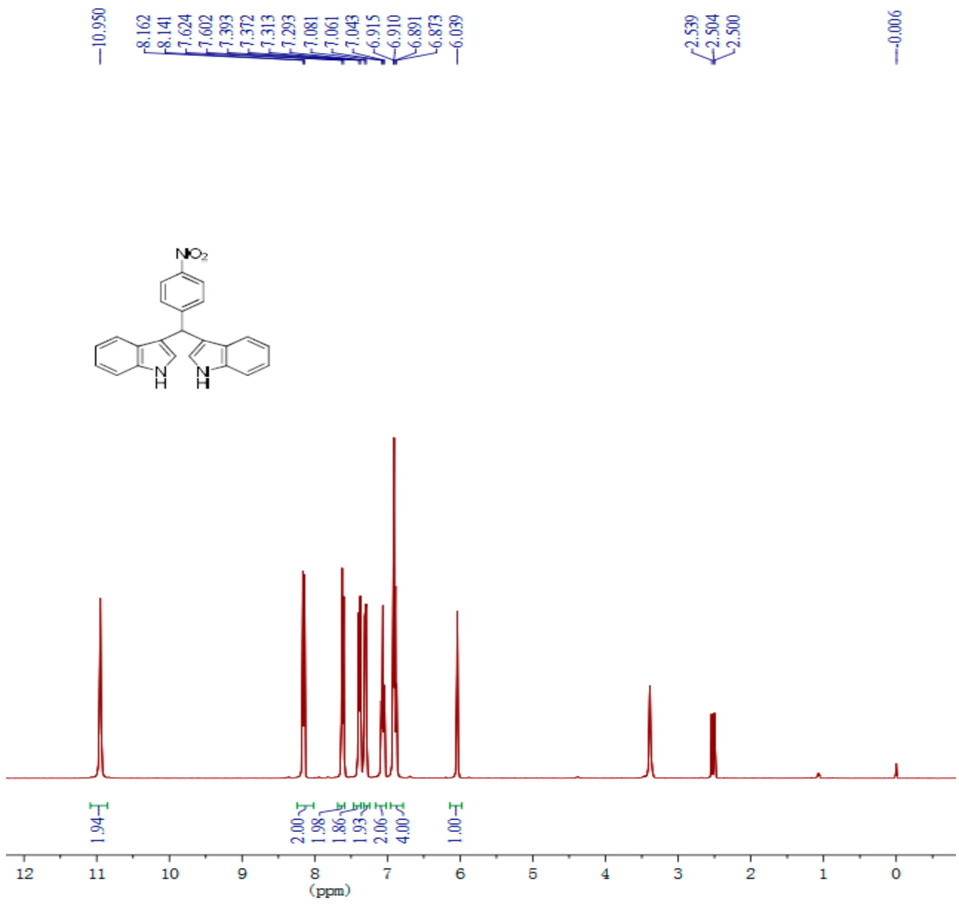

Figure S4. ^{13}C-NMR of compound **3a**.

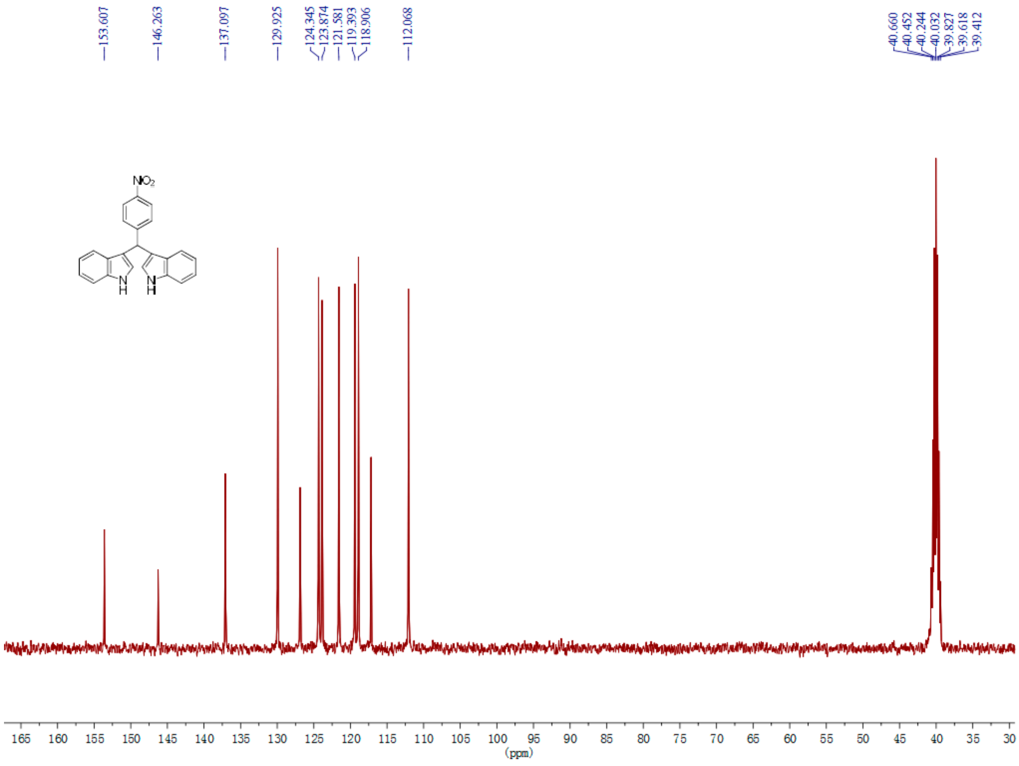

3,3'-((3-Nitrophenyl)methylene)bis(1H-indole) (3b)

Figure S5. IR of compound **3b**.

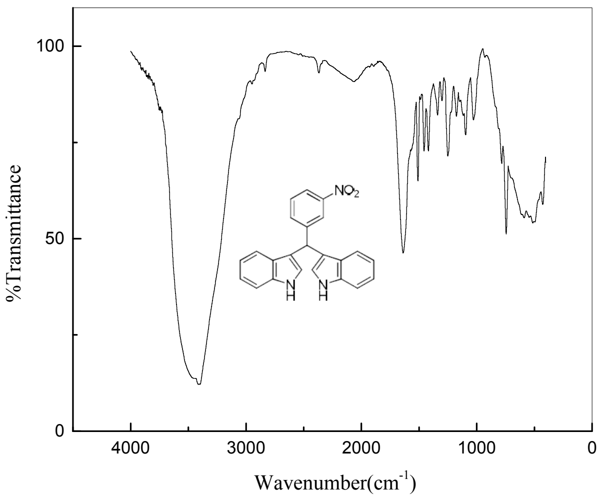

Figure S6. ^{1}H-NMR of compound **3b**.

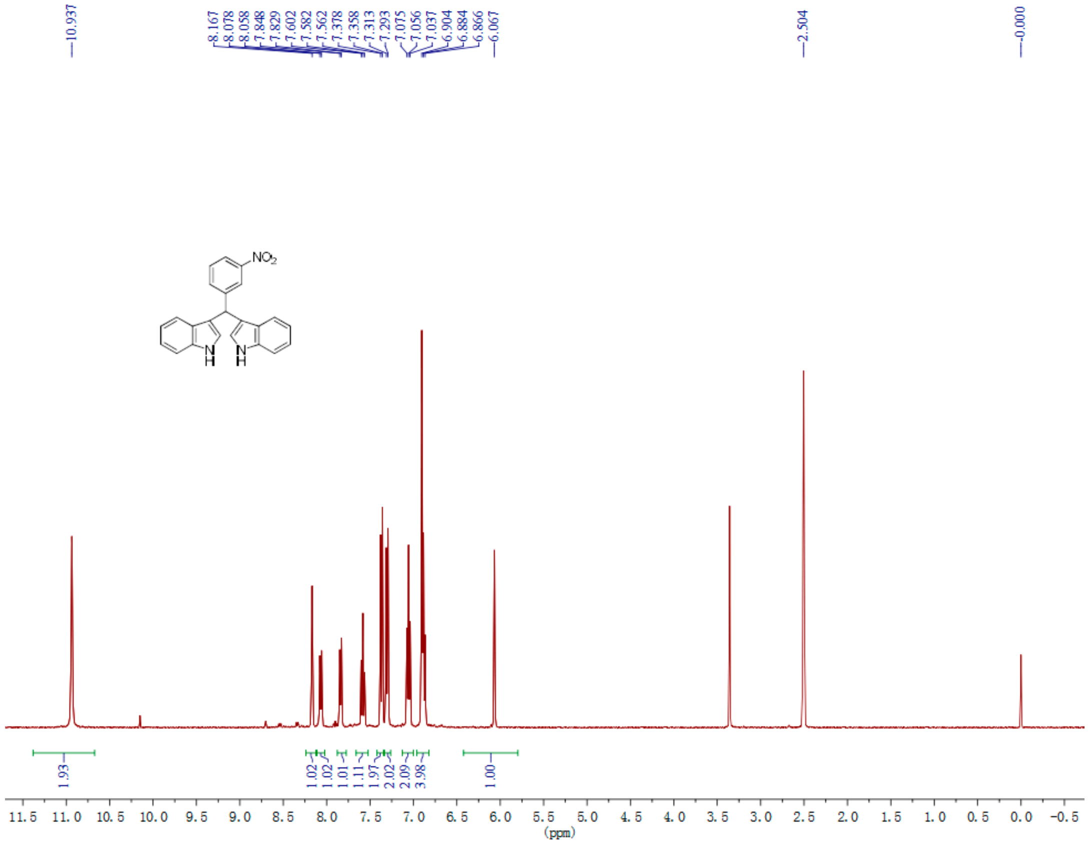

3,3'-((2-Nitrophenyl)methylene)bis(1H-indole) (3c)

Figure S7. IR of compound **3c**.

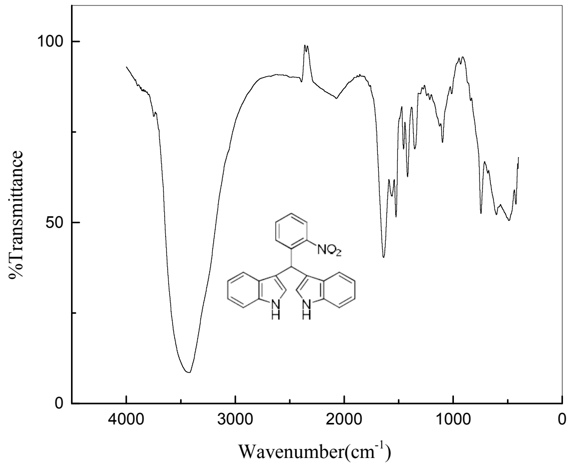

Figure S8. ^{1}H-NMR of compound **3c**.

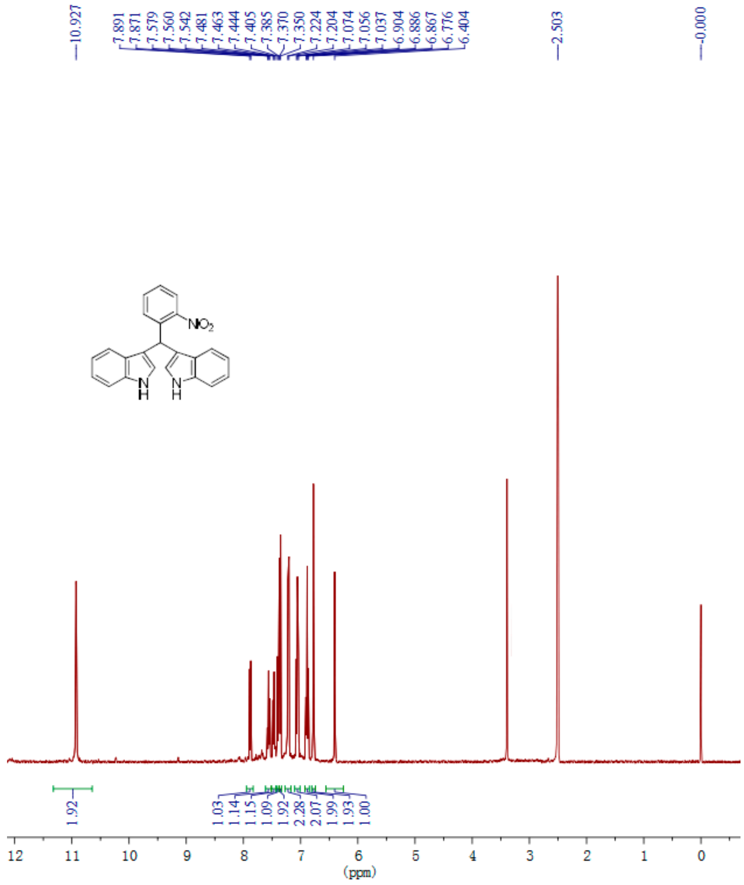

3,3'-((4-Chlorophenyl)methylene)bis(1H-indole) (3d)

Figure S9. IR of compound **3d**.

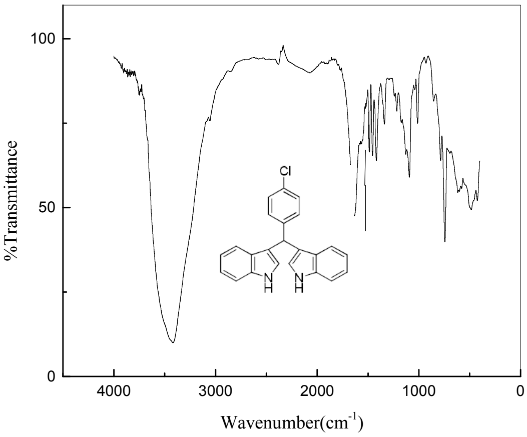

Figure S10. ^{1}H-NMR of compound **3d**.

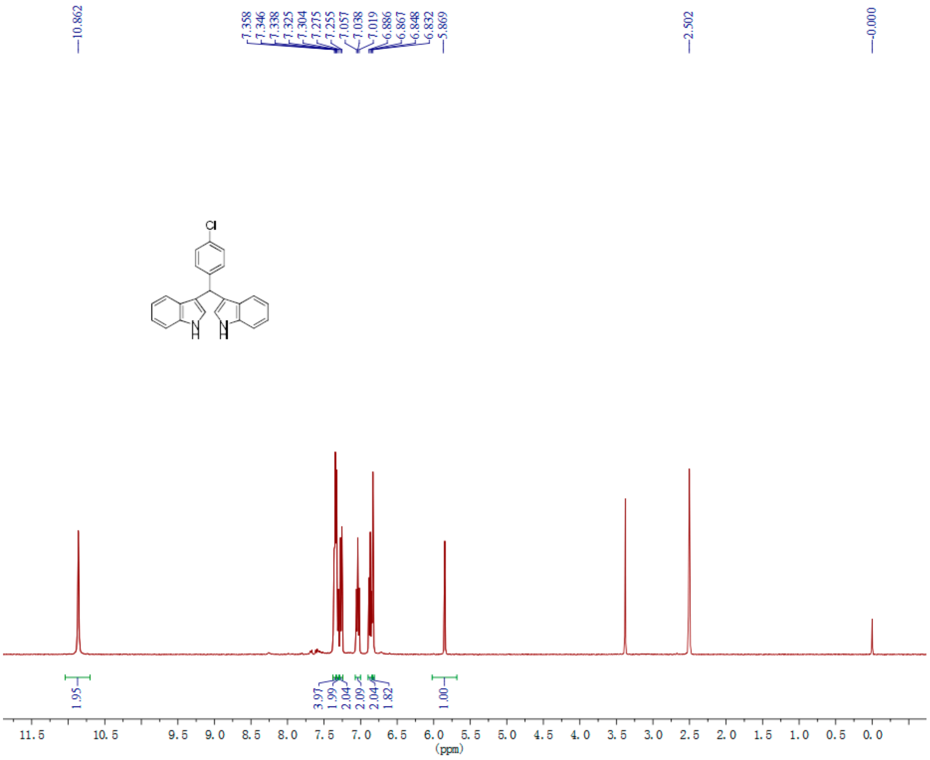

3,3'-((2-Chlorophenyl)methylene)bis(1H-indole) (3e)

Figure S11. IR of compound **3e**.

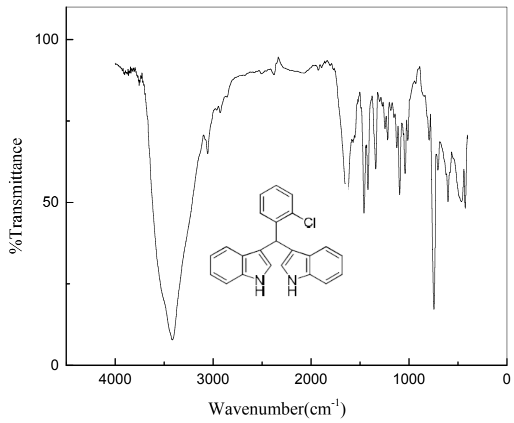

Figure S12. ^{1}H-NMR of compound **3e**.

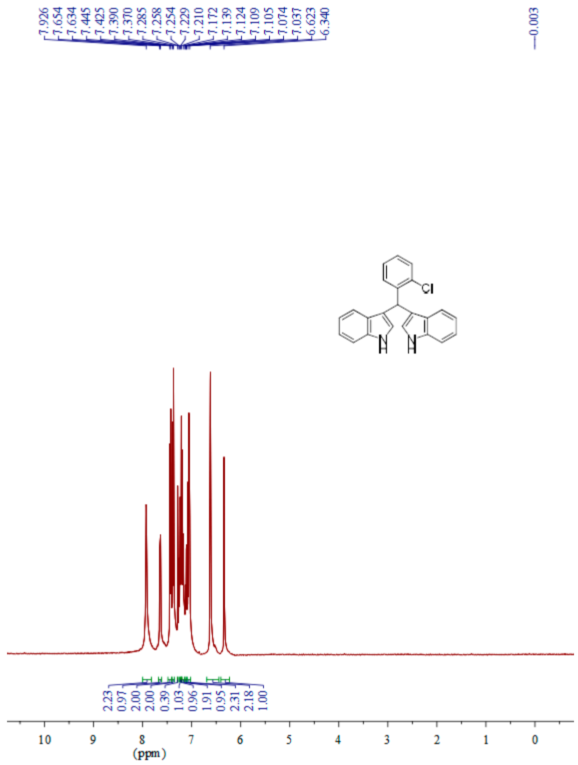

3,3'-((3-Bromophenyl)methylene)bis(1H-indole) (3f)

Figure S13. IR of compound **3f**.

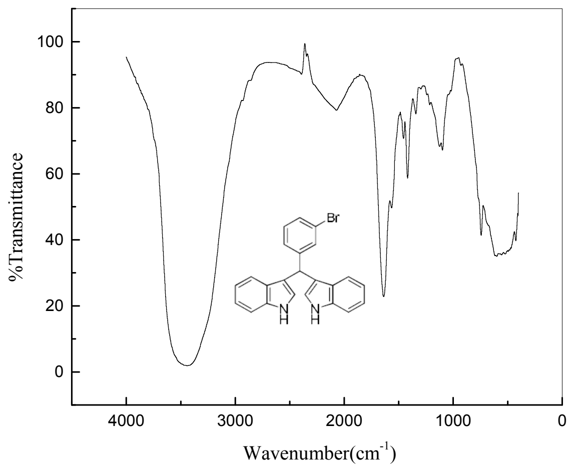

Figure S14. ^{1}H-NMR of compound **3f**.

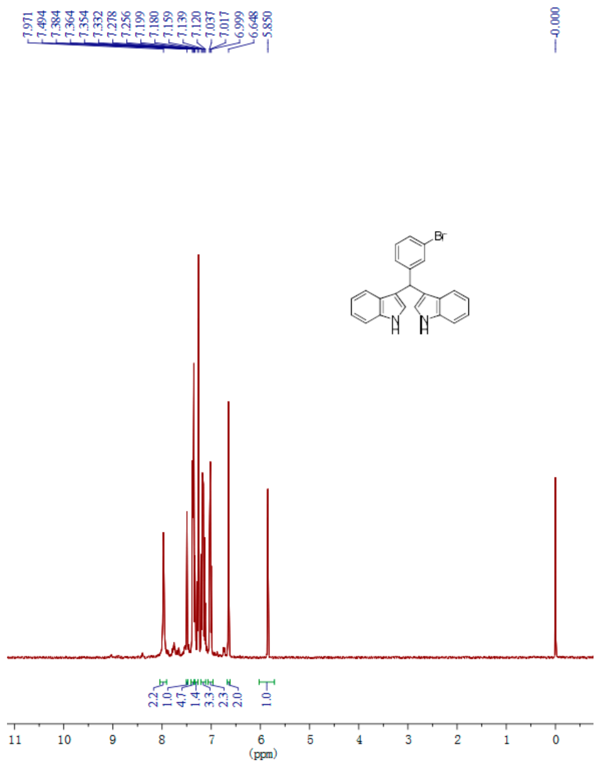

3,3'-((2-Bromophenyl)methylene)bis(1H-indole) (3g)

Figure S15. HRMS of compound **3g**.

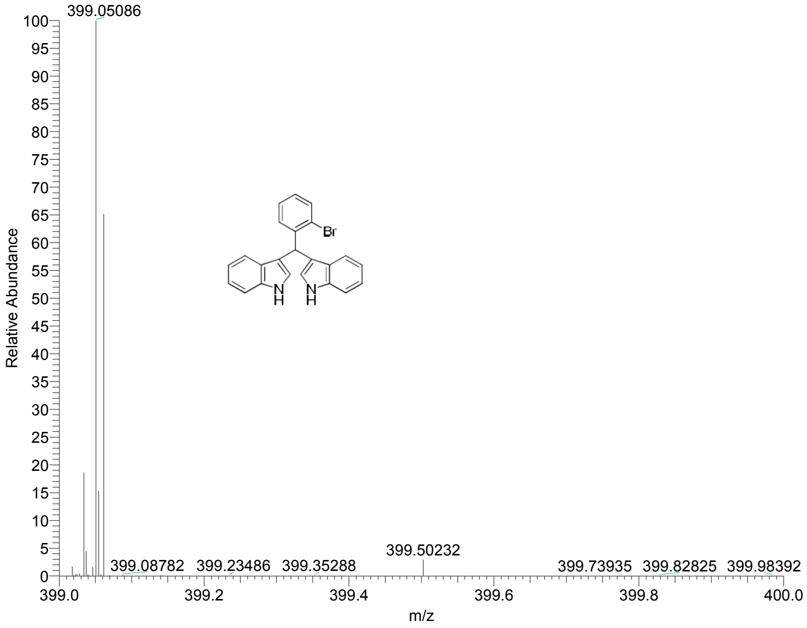

Figure S16. IR of compound **3g**.

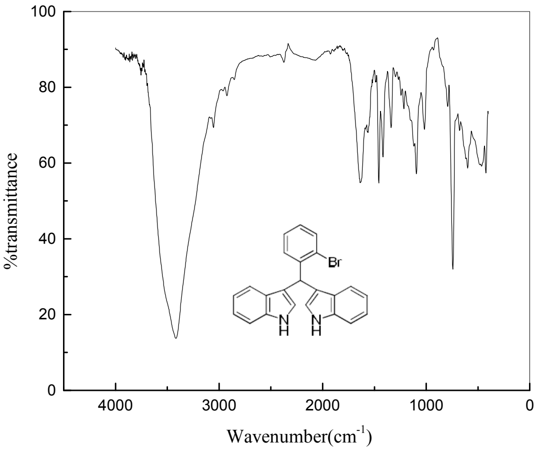

Figure S17. ^{1}H-NMR of compound **3g**.

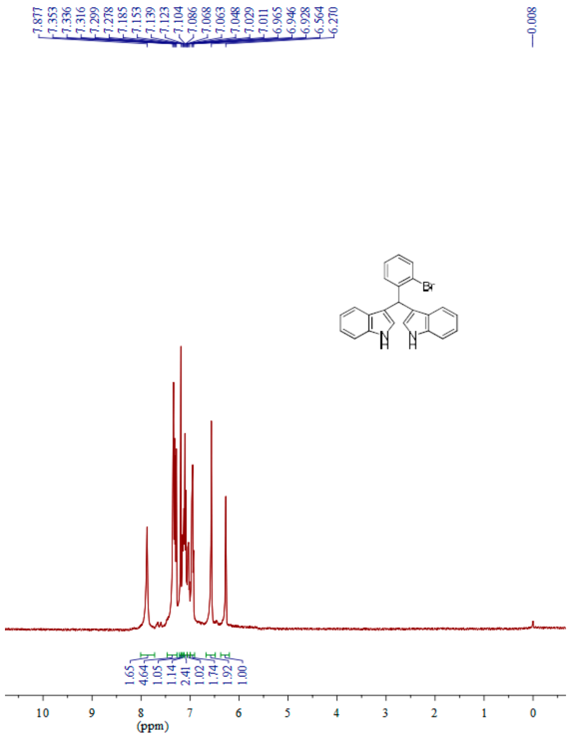

3,3'-((4-Hydroxylphenyl)methylene)bis(1H-indole) (3h)

Figure S18. IR of compound **3h**.

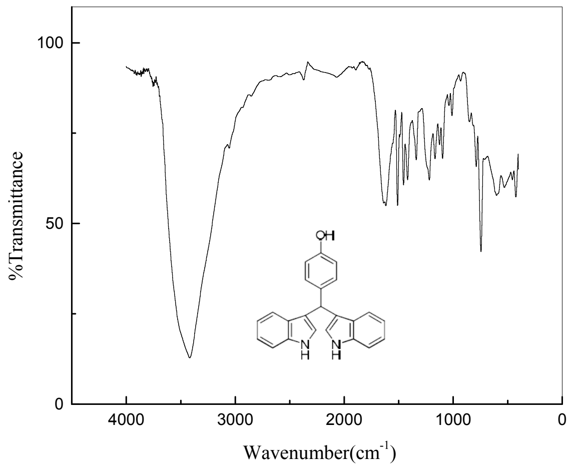

Figure S19. ^{1}H-NMR of compound **3h**.

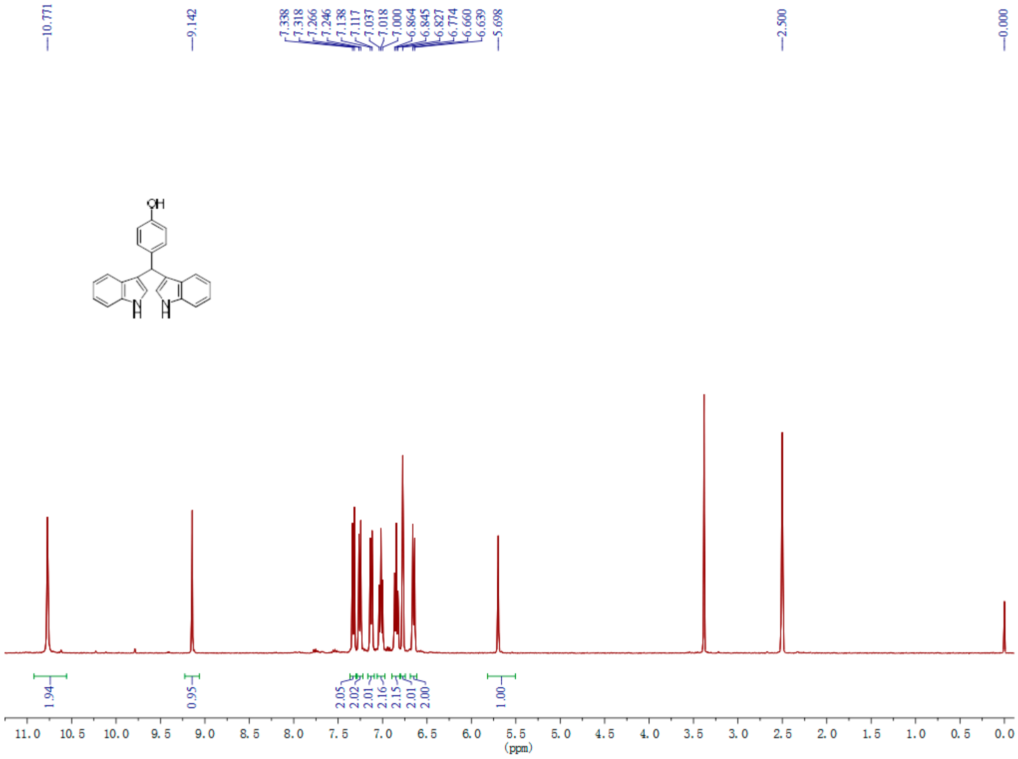

3,3'-((2-Hydroxylphenyl)methylene)bis(1H-indole) (3i)

Figure S20. IR of compound **3i**.

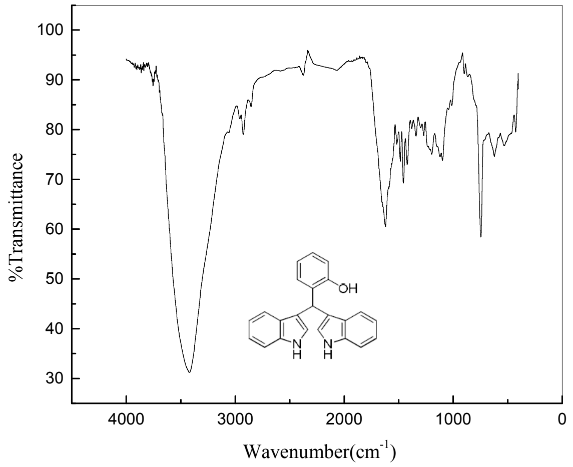

Figure S21. ^{1}H-NMR of compound 3i.

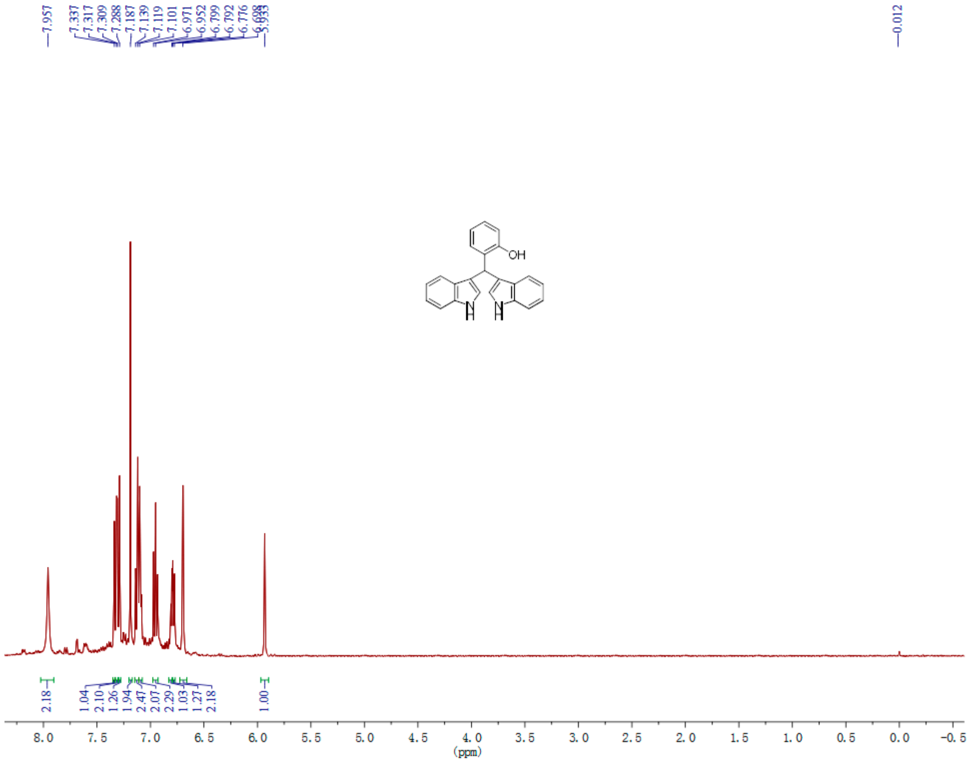

3,3'-((4-Methylphenyl)methylene)bis(1H-indole) (3j)

Figure S22. IR of compound **3j**.

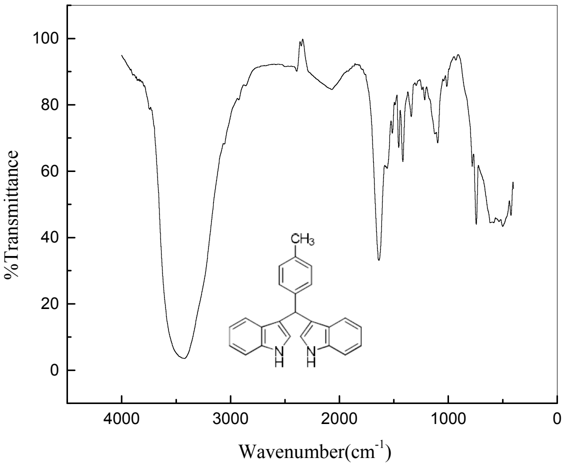

Figure S23. ^{1}H-NMR of compound **3j**.

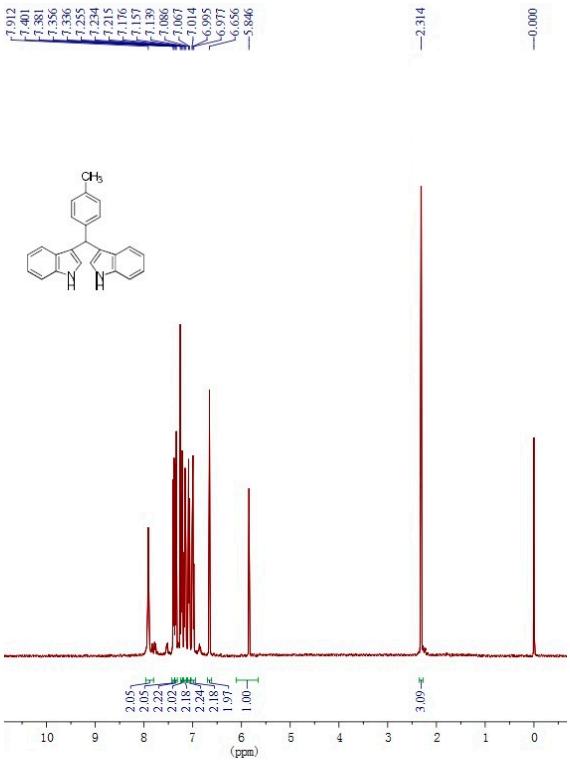

3,3'-((4-Methoxylphenyl)methylene)bis(1H-indole) (3k)

Figure S24. IR of compound **3k**.

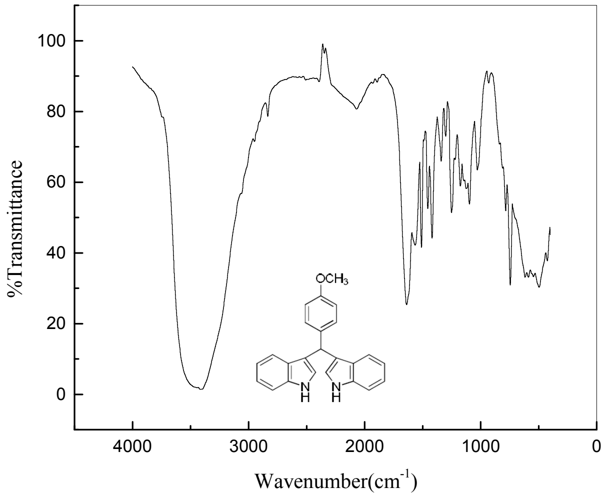

Figure S25. ^{1}H-NMR of compound **3k**.

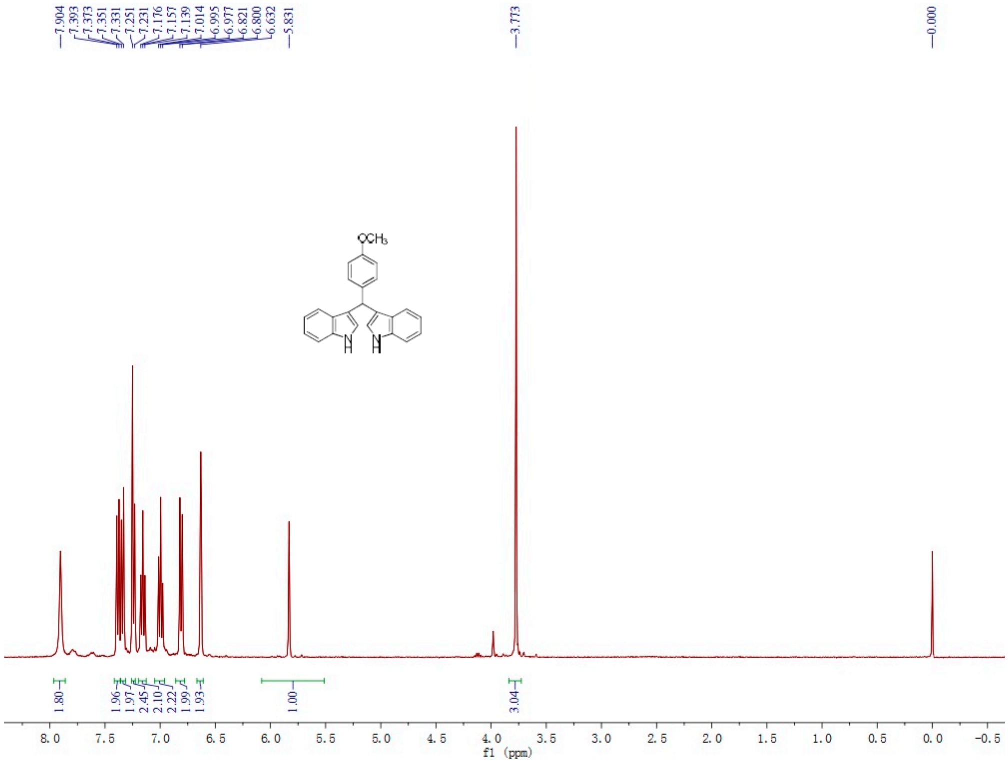

4-(Di(1H-indol-3-yl)methyl)-2-methoxyphenol (3l)

Figure S26. IR of compound **3l**.

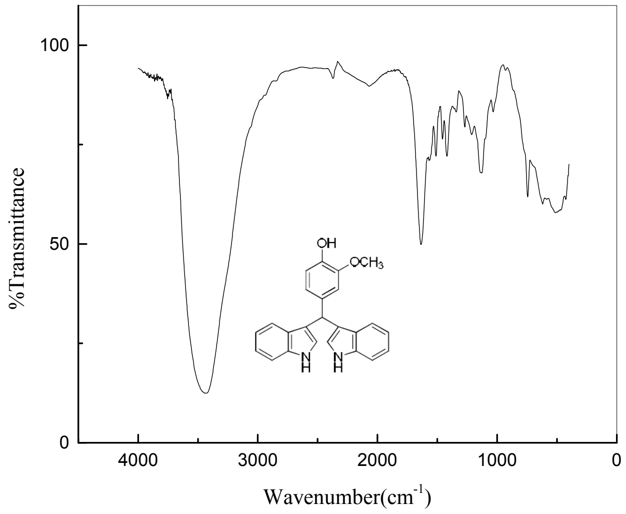

Figure S27. ^{1}H-NMR of compound **3l**.

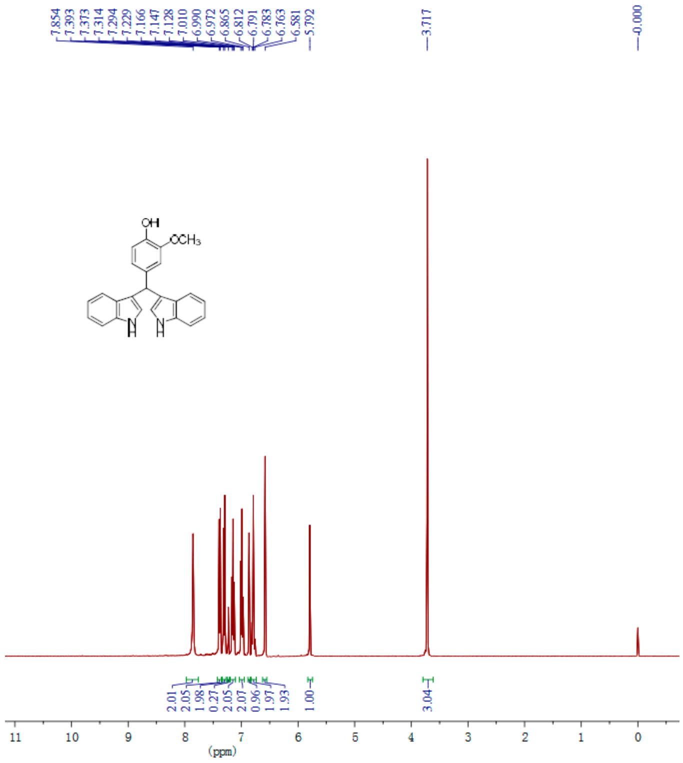

Supplement: Supplementary file 1 [file molecules-19-19665-s001.pdf]
